# Complex modulation of cytokine-induced α-synuclein aggregation by glypican-1-derived heparan sulfate in neural cells

**DOI:** 10.1093/glycob/cwab126

**Published:** 2021-12-07

**Authors:** Fang Cheng, Lars-Åke Fransson, Katrin Mani

**Affiliations:** Department of Experimental Medical Science, Division of Neuroscience, Glycobiology Group, Lund University, Biomedical Center A13, SE-221 84 Lund, Sweden; Department of Experimental Medical Science, Division of Neuroscience, Glycobiology Group, Lund University, Biomedical Center A13, SE-221 84 Lund, Sweden; Department of Experimental Medical Science, Division of Neuroscience, Glycobiology Group, Lund University, Biomedical Center A13, SE-221 84 Lund, Sweden

**Keywords:** glypican-1, heparan sulfate, nitric oxide, Parkinson’s disease, α-synuclein

## Abstract

In Parkinson’s disease (PD), there is accumulation of α-synuclein (SYN) aggregates in neurons, which is promoted by neuroinflammation. The cytokines TNF-α, IL-1β and IL-6 induce accumulation of degradation products of the amyloid precursor protein (APP) combined with heparan sulfate (HS) chains released from glypican-1 (Gpc-1) by NO-dependent cleavage. We have investigated the effects of the cytokines and HS on SYN aggregation and secretion in dividing human neuroblastoma (SH-SY5Y) and inducible neural progenitor cells (NPC) by using immunofluorescence microscopy, vesicle isolation and slot blotting with antibodies recognizing SYN monomers and aggregates, Gpc-1, the released HS, endosomes, and autophagosomes. In SH-SY5Y cells, the capacity to release HS was fully utilized, while NPC displayed dormant capacity. TNF-α induced increased formation of SYN aggregates and clustering of HS in SH-SY5Y cells. When the supply of NO was simultaneously increased, SYN and HS accumulation disappeared. When NO formation was inhibited, SYN and HS aggregation also disappeared, but there was now a 4-fold increase in SYN secretion. In NPC, IL-6 induced increased aggregation of SYN and stimulated HS release from Gpc-1. Both SYN and HS co-localized with autophagosome marker. When HS-deficient Gpc-1 was simultaneously generated, by using a cyanobacterial neurotoxin, accumulation diminished and there was massive secretion of SYN. We suggest that the cytokines increase APP processing, which initiates NO-dependent release of HS from Gpc-1. The APP degradation products also trigger SYN aggregation. As HS can inhibit APP processing, HS- or NO-deficiency may result in autophagosomal dysfunction and both APP degradation products and SYN are secreted.

## Introduction

**Fig. 1:**
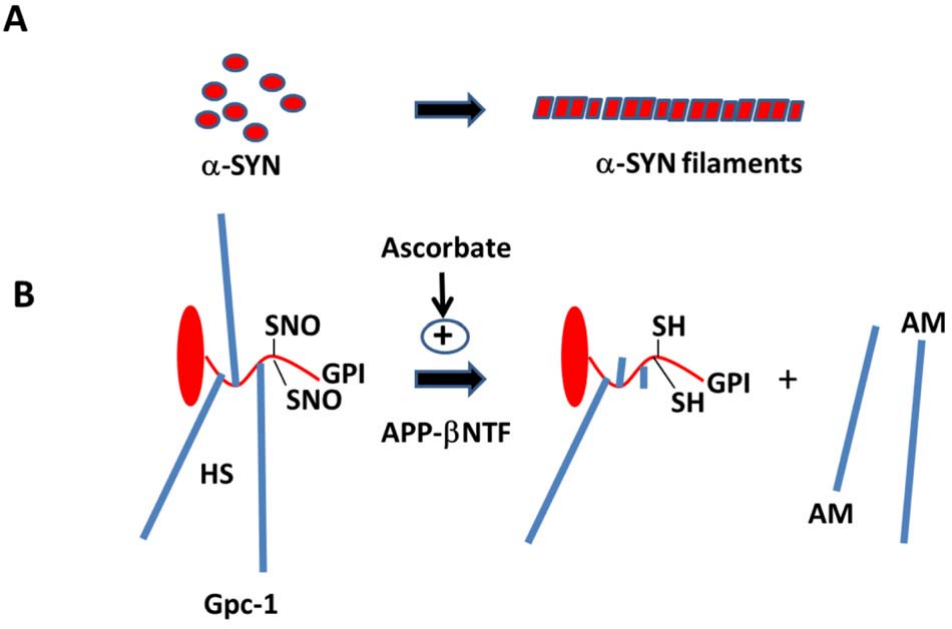
Aggregation of α-SYN to α-SYN filaments and NO-dependent deaminative release of HS from Gpc-1. (A) Conformational changes in the N-terminal domain of α-SYN monomers can induce filamentous aggregation. (B) The major part of the Gpc-1 protein (red) consists of an elongated N-terminal domain followed by a smaller flexible segment that is substituted with three HS chains (blue), contains two SNO cysteines and terminates with a GPI lipid anchor. The SNO groups participate in the deaminative release of HS, which is catalyzed by the N-terminal, β-secretase-released domain of the amyloid precursor protein (APP-βNTF) and is stimulated by ascorbate (vitamin C). The released HS chains contain reducing terminal anhydromannose (AM). SH, cysteine thiols.

Parkinson disease (PD) is the second most prevalent neurodegenerative disorder after Alzheimer disease (AD). In PD, there is a significant loss of dopaminergic neurons and accumulation of intracytoplasmic aggregates composed mainly of α-synuclein (SYN). There is a causal role for SYN aggregation in the pathogenesis of both familial and sporadic PD. SYN is a 140 amino acid protein, highly expressed in neural cells, but with a poorly understood function. It is soluble and resides in the cytoplasm, often located to presynaptic nerve terminals and occasionally at the nuclear envelope (Maroteaux et al. 1988). SYN can also bind to the cytosolic surface of intracellular membranes, especially to so-called lipid rafts. Binding to these rafts alters the conformation of the N-terminal domain of SYN, which facilitates filament formation (Fig. 1A). Clearance of SYN monomers as well as oligomers occurs by autophagy and lysosomal degradation or by secretion via exosome-like vesicles. A fragment of SYN (61–95) accumulates together with amyloid beta (Aβ) in AD plaques (for reviews, see e.g. Bendor et al. 2013; Boya et al. 2013; Guardia-Laguarta et al. 2015; Budnik et al. 2016; Hessvik and Llorente 2018).

Proteins that contain a C-terminal glycosyl-phosphatidyl-inositol (GPI) lipid anchor are typically concentrated to lipid rafts. One example is the widely expressed heparan sulfate (HS)-containing and recycling, cell-surface proteoglycan glypican-1 (Gpc-1), which becomes S-nitrosylated (SNO) during caveolin/lipid raft-dependent endocytosis (Cheng et al. 2002; Watanabe et al. 2004). Gpc-1 and the amyloid precursor protein (APP), which is involved in AD, bind strongly to one another and are both processed in endosomes (Reinhard et al. 2005; Mani et al. 2006). Gpc-1 releases its HS chains by an intrinsic SNO-catalyzed deaminative cleavage generating HS chains and oligosaccharides with a reducing terminal anhydromannose (HS-anMan). This reaction is initiated by the N-terminal fragment of APP (β-NTF), which is stimulated by ascorbate (Fig. 1B). HS-anMan is then transported into the cytosol (Mani et al. 2006; Cheng et al. 2011, 2012, 2014, 2017).

The pathogenesis of PD may be due to neurotoxic effects of filamentous SYN aggregates in the nucleus and mitochondria or to autophagosomal dysfunction, and can be promoted by neuroinflammation. In advanced stages of PD, there is often also cognitive decline. Excessive, involuntary dietary intake of the cyanobacterial neurotoxin β-N-methylamino-L-alanine (BMAA) is believed to be the cause of a dementia with features of both PD and AD (for reviews, see Cox and Sacks 2002; Guardia-Laguarta et al. 2015; Aarsland et al. 2017; Cox et al. 2018; Joshi and Singh 2018; Rocha et al. 2018; Vasquez et al. 2020).

We have recently reported that the proinflammatory cytokines tumor necrosis factor-alpha (TNF-α), interleukin 1-beta (IL-1β) and interleukin-6 (IL-6), which stimulate β-cleavage of APP, induce accumulation of Gpc-1-derived HS-anMan and the C-terminal fragment of APP (β-CTF) in enlarged autophagosomes of dividing neuronal cells. Accumulation was modulated by up- or down-regulation of HS-anMan release (Cheng et al. 2020). Here, we show a similar effect on SYN aggregation and secretion in dividing human neuroblastoma and inducible neural progenitor cells.

## Results

### α-SYN, Gpc-1 and deaminatively released heparan sulfate (HS-anMan) co-localize in human neuroblastoma (SH-SY5Y) cells

**Fig. 2:**
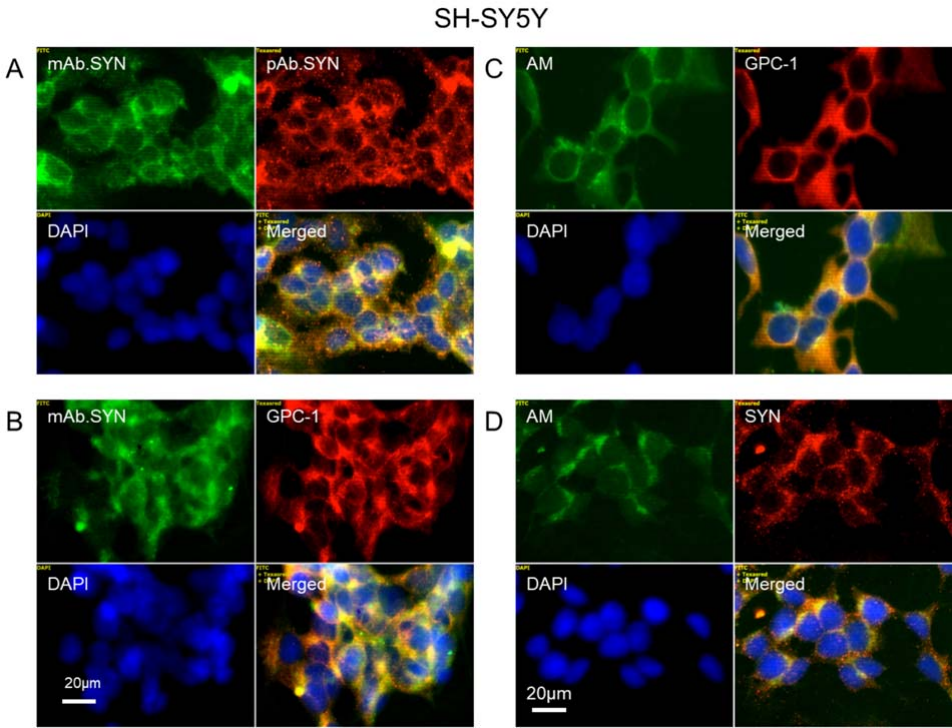
α-SYN co-localizes with Gpc-1 and AM-containing heparan sulfate (HS-anMan) in the cytoplasm of human neuroblastoma SH-SY5Y cells. Representative immunofluorescence images of cell cultures grown to near confluence in regular medium. Staining was performed with mAb SYN (green), pAb SYN (red), pAb GPC-1 (red), mAb AM (for HS-anMan) and DAPI (for nuclei, blue). Exposure time was the same in all cases. Bar, 20 μm.

We demonstrated the presence of SYN, Gpc-1 and HS-anMan in SH-SY5Y cells by deconvolution immunofluorescence microscopy using both mAb SYN and pAb SYN, pAb GPC-1 and mAb AM (specific for HS-anMan; Pejler et al. 1988). Staining for SYN by the two different antibodies co-localized in the cytoplasm of the SH-SY5Y cells, as expected (Figs 2A and Supplementary Figure S1A, yellow in merged). mAb SYN and pAb GPC-1 staining also co-localized partially in the cytoplasm (Figs 2B and Supplementary Figure S1B). There was strong co-localization between HS-anMan (AM) and Gpc-1 staining (Figs 2C and Supplementary Figure S1C, merged), and partial co-localization between HS-anMan (AM) and SYN (Figs 2D and Supplementary Figure S1D, merged). These results indicate, firstly, that spontaneous, deaminative release of HS from Gpc-1 is active in these cells. Secondly, Gpc-1, HS-anMan and SYN can be closely located to one another.

### Proinflammatory cytokines increase α-SYN filament accumulation in SH-SY5Y cells

SYN filamentous (SYNfil) aggregates were detected by immunofluorescence microscopy using a conformation-specific, rabbit mAb. Low magnification images showed staining for SYNfil in untreated cells (Supplementary Figure S2A, SYNfil, green). In a few cases, it appeared separately in clusters (Supplementary Figure S2A, SYNfil, green in merged). When cells were grown to confluence in the presence of the cytokines TNF-α, IL-1β, or IL-6, there was strong co-localization between SYNfil and SYN (Supplementary Figures S2B, C, and D, SYNfil, green and yellow in merged).

**Fig. 3:**
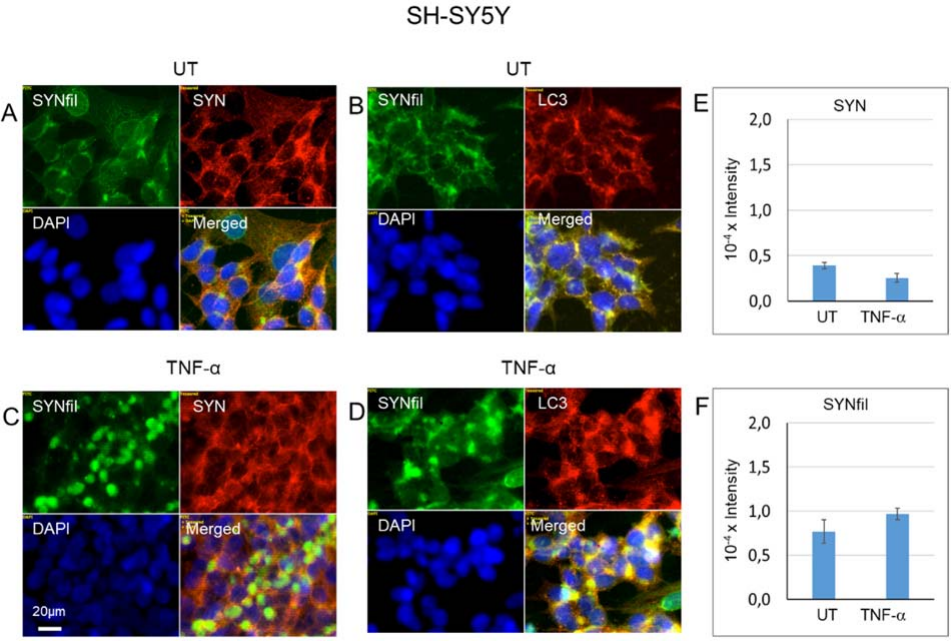
TNF-α induces accumulation of α-SYNfils in SH-SY5Y cells. Representative immunofluorescence images of SH-SY5Y cells which were grown to confluence in regular medium (A, B, UT = untreated) or in medium containing 100 pg/ml TNF-α (C, D). Staining was performed with mAb SYNfil (green), pAb SYN (red), a pAb against LC3 (autophagosome marker, red) and DAPI (for nuclei, blue). Exposure time was the same in all cases. Bar, 20 μm. Slot blot assay for SYN (E) and SYNfil (F) in lysates of LC3-positive vesicles (total volume, 1 ml; sample volume, 30 μL). The intensity of the controls (no primary antibody added) was subtracted. The error bars indicate S.E., *n* = 5.

When untreated and TNF-α-treated cells were also examined at higher magnification (Fig. 3), increased SYNfil cluster formation was observed in cytokine-treated cells (cf. Fig. 3A and B with Fig. 3C and D, SYNfil, green). The clusters stained almost exclusively for SYNfil in the center (Fig. 3C, green in merged), while co-localization with SYN was present at the periphery of the clusters (Fig. 3C, yellow in merged). We also co-stained with a pAb against LC3, a marker for autophagosomes. In untreated cells, SYNfil co-localized with LC3 (Fig. 3B, yellow in merged), while the TNF-α-induced SYNfil clusters co-localized partially with LC3, indicating accumulation both in the cytosol and in autophagosomes (Fig. 3D, green and yellow in merged). A slight enlargement of the autophagosomes was observed (Fig. 3D, LC3, red). We also performed immunomagnet isolation of LC3-positive vesicles from sucrose homogenates of untreated and TNF-α-treated cells, followed by lysis and assay for SYN and SYNfil by slot blotting. The cytokine treatment did not increase the SYN/SYNfil content in these vesicles, indicating that the SYNfil clusters were in the cytosol (Fig. 3E and F).

When SH-SY5Y cells grown in the presence of IL-1β or IL-6 were examined at high magnification, the increased SYNfil staining was partly clustered in IL-1β-treated cells (Supplementary Figure S3A, yellow and separate green in merged), but mostly uniformly co-localized with SYN staining in IL-6-treated cells (Fig. S3B, yellow in merged).

As shown previously, TNF-α stimulates increased processing of APP in growing mouse N2a neuroblastoma and human neural stem cells, whereby the released β-NTF stimulates formation of HS-anMan, which accumulates in enlarged autophagosomes (Cheng et al. 2020). Therefore, we also stained TNF-α-treated SH-SY5Y cells with mAb AM and pAb LC3. In untreated cells, there was partial co-localization between HS-anMan and LC3 staining (Supplementary Figure S4A, green and yellow in merged). In TNF-α-treated cells, the HS-anMan staining was more clustered (Supplementary Figure S4B, AM, green). The extent of co-localization of HS-anMan and the autophagosome marker LC3 in untreated and TNF-α-treated cells appeared to be the same (Supplementary Figure S4A and B, merged; Pearson R value for G:R was approx. 1 in both cases). Taken together, this suggests that the capacity to generate HS-anMan was exhausted in SH-SY5Y cells and that autophagosomal accumulation of SYN and HS-anMan was limited.

### Altered α-SYN filament (SYNfil) accumulation and α-SYN secretion when release of heparan sulfate (HS-anMan) from Gpc-1 is modulated in TNF-α-treated SH-SY5Y cells

**Fig. 4:**
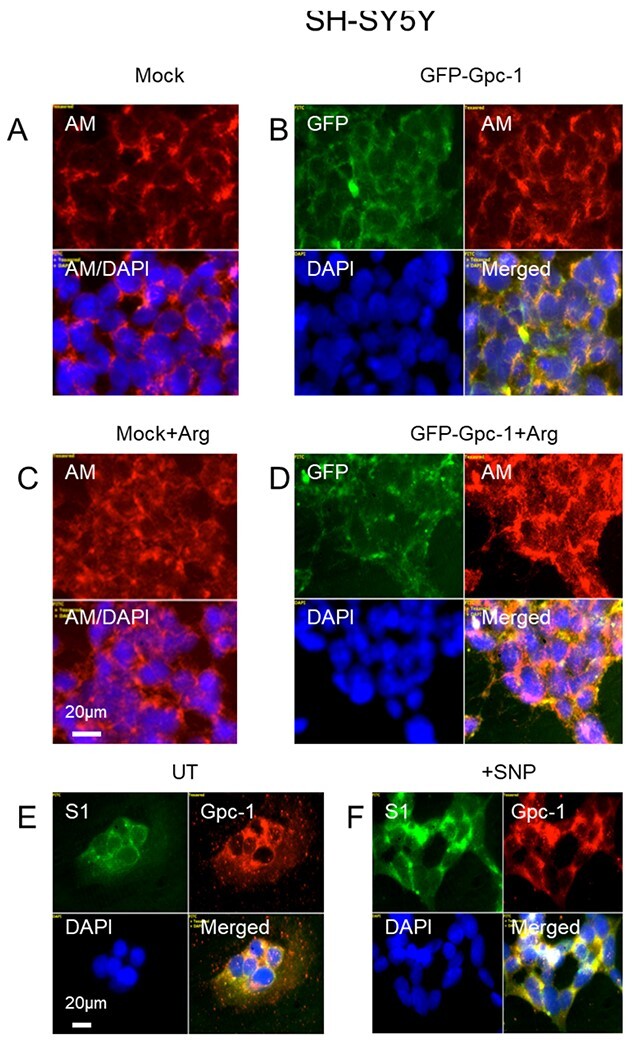
Overexpression of Gpc-1 and supplementation with arginine (Arg) increase the capacity to generate AM-containing heparan sulfate (HS-anMan) in SH-SY5Y cells. Representative immunofluorescence images of SH-SY5Y cells, which were treated with lipofectamine without vector (A, C, mock) or with a vector encoding green fluorescent protein (GFP)-tagged mouse Gpc-1 (B, D) in the absence (A, B) or presence of 1 mM Arg (C, D) or left untreated (E, UT) or exposed to 1 mM sodium nitroprusside (SNP) for 30 min (F). Staining was performed with mAb AM (for HS-anMan, A-D, red; Alexa Fluor 594-tagged secondary antibody), mAb S1 (for Gpc-1-SNO, E-F, green), anti-human Gpc-1 (E-F, red) and DAPI (for nuclei, blue), while GFP-Gpc-1 is visualized in the green channel of A-D. The intensities of HS-anMan and DAPI staining in A-D were measured and expressed as the ratio AM/DAPI. Exposure time was the same in all cases. Bar, 20 μm.

The capacity to generate HS-anMan is dependent on the content of Gpc-1 and the supply of NO. SH-SY5Y cells were transiently transfected with a vector encoding green fluorescent protein (GFP)-tagged mouse Gpc-1 protein. GFP-Gpc-1 was expressed and recycled to Rab7-positive late endosomes (Supplementary Figure S4C, yellow in merged). Transfection with GFP-Gpc-1 increased the HS-anMan staining intensity by approximately 50% compared with mock transfected cells, indicating that GFP-Gpc-1 carried HS chains (Fig. 4A, AM/DAPI 0.61; Fig. 4B, AM/DAPI 0.92). Release of HS-anMan from the Gpc-1 proteoglycan is a deaminative process that requires continuous supply of NO (Cheng et al. 2012). NO is generated by NO-synthases from the amino acid arginine (Arg) and molecular oxygen (O_2_) (Stamler et al. 1992). We therefore supplemented the transfection media with 1 mM Arg. This resulted in an approximate doubling of HS-anMan staining intensity in mock transfected cells (Fig. 4A, AM/DAPI 0.61; Fig. 4C, AM/DAPI 1.16). The combined effect of GFP-Gpc-1 transfection and Arg supplementation also resulted in a similar increase in HS-anMan staining intensity (Fig. 4A, AM/DAPI 0.61; Fig. 4D, AM/DAPI 1.21), suggesting that the supply of NO was the limiting factor. The reason for this could be a low level of Gpc-1 S-nitrosylation in the untreated cells. This was confirmed by using mAb S1, which recognizes Gpc-1-SNO (Cheng et al. 2002; Mani et al. 2003). There was a marked increase in S1 staining intensity when cells were exposed to an NO-donor (cf. Figs 4E and F, S1 green, increased yellow in merged).

**Fig. 5:**
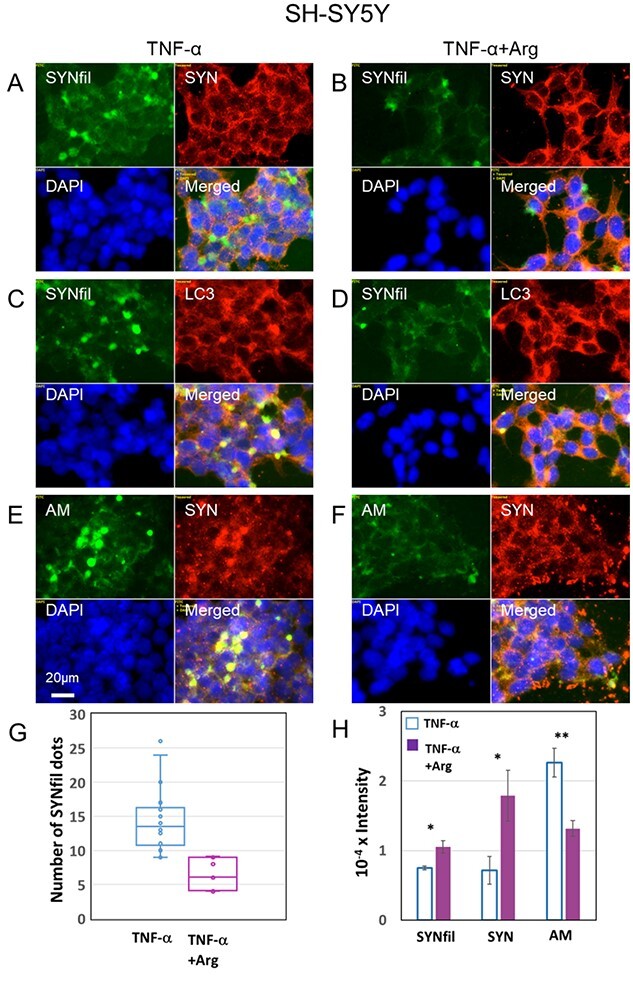
Supplementation with arginine (Arg) reduces filamentous aggregation and autophagosomal accumulation of α-SYN (SYNfil) in tumor necrosis factor-α (TNF-α)-treated SH-SY5Y cells. Representative immunofluorescence images of SH-SY5Y cells, which were grown to confluence in medium containing 100 pg/ml TNF-α (a, C, E) or 100 pg/ml TNF-α and 1 mM Arg (B, D, F). Staining was performed with mAb SYNfil (green), pAb SYN (red), mAb AM (for HS-anMan, green), a pAb against LC3 (autophagosomes marker, red) and DAPI (for nuclei, blue). Exposure time was the same in all cases. Bar, 20 μm. (G) Whisker plot of the number of SYNfil clusters in individual images from repeated experiments with SH-SY5Y cells grown in the presence of TNF-α (*n* = 16) or TNF-α and Arg (*n* = 9). (H) Slot blot assay of SYNfil, SYN and HS-anMan (AM) secretion into the media (total volume, 2.8 ml) of cell cultures grown to confluence in the presence of TNF-α or TNF-α and Arg. Sample volume, 30 μL. The intensity of the controls (no primary antibody added) was subtracted. The error bars indicate S.E., *n* = 5; ^*^^*^, *P* < 0.01.

TNF-α induced increased SYNfil cluster formation in growing SH-SY5Y cells (Fig. 3C and D, green). To examine the effect of Arg on SYN aggregation, SH-SY5Y cells were grown to confluence in the presence of both TNF-α and Arg. SYNfil formation and clustering was reduced when 1 mM Arg was included in the growth medium (cf. Fig. 5A and C with Fig. 5B and D, green and merged). The intensity of the SYN staining was unaffected by supplementation with Arg (cf. Fig. 5A and E with Fig. 5B and F, red). In contrast, the HS-anMan staining, which co-clustered with SYN (Fig. 5E, green and yellow in merged) almost disappeared when Arg was included in the growth medium (Fig. 5F, green). We also made quantitative estimates of the effect of Arg on SYNfil clustering. Counting SYNfil clusters in individual images from repeated experiments indicated, on an average, approx. 50% reduction of clusters in the presence of Arg (Fig. 5G). As accumulated SYNfil and HS-anMan can either be degraded in lysosomes or secreted from autophagosomes into the medium, we also assayed the media for SYNfil, SYN and HS-anMan (AM) by slot blotting. The results showed that there was essentially no effect of Arg on aggregated SYN (SYNfil) secretion, a doubling of total SYN secretion, and a decrease in HS-anMan secretion (Fig. 5H). As the cellular content of SYN appeared unchanged and as there was no excessive secretion of SYN, the disappearance of SYNfil/HS-anMan clustering could be due to disaggregation and/or degradation of SYNfil and HS-anMan.

**Fig. 6:**
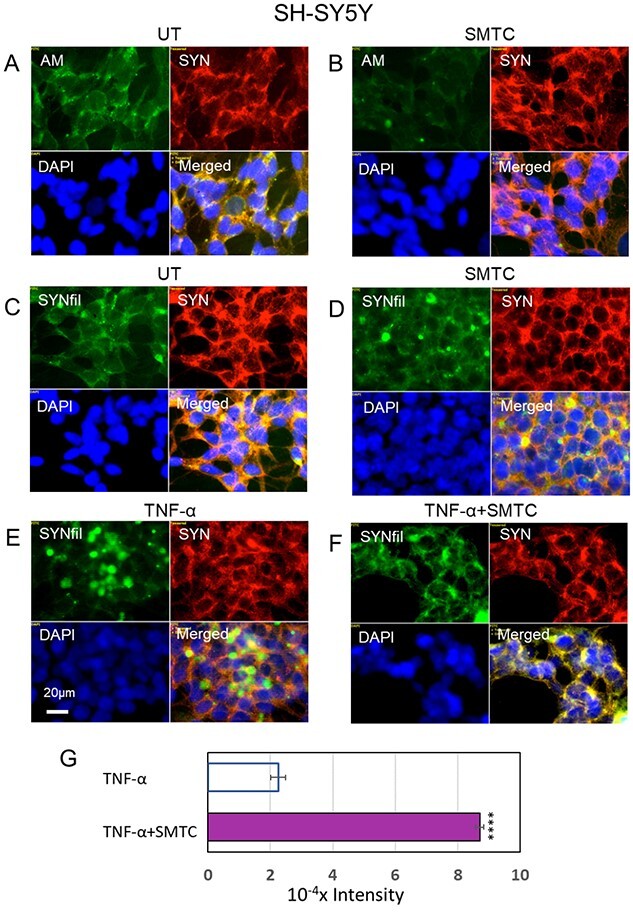
Inhibition of neural NO-synthase diminishes release of AM-containing HS (HS-anMan) from glypican-1 (Gpc-1), prevents TNF-α-induced clustering of α-SYNfils in SH-SY5Y cells and increases α-SYN secretion. Representative immunofluorescence images of SH-SY5Y cells which were grown to confluence in regular medium (A, C; UT = untreated), or in medium containing 100 μM S-methyl-L-thiocitrulline (B, D; SMTC) or 100 pg/ml tumor necrosis factor-α (E, TNF-α) or both (F, TNF-α + SMTC). Staining was performed with mAb AM (for HS-anMan, green), pAb SYN (red), mAb SYNfil (green) and DAPI (for nuclei, blue). Exposure time was the same in all cases. Bar, 20 μm. (G) Slot blot assay of SYN secretion into the media (total volume, 2.8 ml) of cell cultures grown to confluence in the presence of TNF-α or TNF-α and SMTC. Sample volume: 30 μL. The intensity of the controls (no primary antibody added) was subtracted. The error bars indicate S.E., *n* = 5; ^*^^*^^*^^*^, *P* < 0.0001.

To explore the effect of NO-deprivation on HS-anMan formation and SYNfil clustering, SH-SY5Y cells were grown to confluence in medium containing the selective neural NO-synthase inhibitor S-methyl-L-thiocitrulline (SMTC) (Melikian et al. 2009). This resulted in decreased overall staining intensity for HS-anMan, while a few clusters remained (Fig. 6A and B, AM, green). The overall staining intensity for SYNfil was not appreciably altered, but a few clusters of SYNfil co-localizing with SYN were observed (Fig. 6C and D, green and yellow in merged). In contrast, TNF-α-induced, clustered SYNfil accumulation was greatly reduced in cells grown in the presence of both the cytokine and NO-synthase inhibitor (cf. Figure 6E and F, from green/yellow clusters to diffuse yellow in merged). To assess the extent of secretion we also assayed the media for SYN by slot blotting. There was an almost 4-fold increase in secretion of SYN when HS-anMan formation was suppressed in TNF-α-treated cells (Fig. 6G). Taken together, these results suggest a regulatory role for HS-anMan in SYN aggregation, degradation and clearance.

### Altered α-SYNfil accumulation and α-SYN secretion when release of heparan sulfate (HS-anMan) from Gpc-1 is modulated in IL-6-treated human neural progenitor cells

**Fig. 7:**
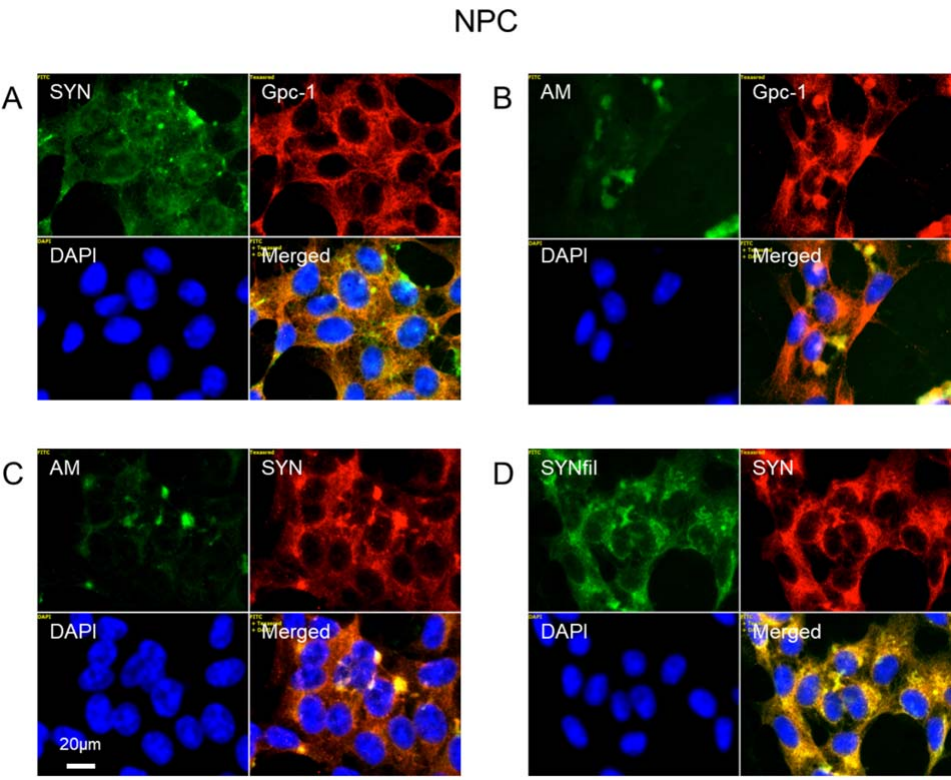
α-SYN, SYNfils and Gpc-1 co-localize in the cytoplasm of human NPC. Representative immunofluorescence images of confluent cultures of NPC. Staining was performed with mAb SYN (A, green), mAb AM (for HS-anMan, B, C, green), mAb SYNfil (D, green), pAb GPC-1 (A, B, red), pAb SYN (C, D, red) and DAPI (for nuclei, blue). Exposure time was the same in all cases. Bar, 20 μm.

In neural progenitor cells (NPC), staining for SYN and Gpc-1 showed partial co-localization throughout the cytoplasm (Fig. 7A, orange in merged). Staining for HS-anMan appeared in a few cytoplasmic clusters of some cells, where it co-localized with staining for Gpc-1 (Fig. 7B, yellow in merged). However, in many cells staining for HS-anMan was very weak (Fig. 7B and C, AM, green; DAPI, blue). This suggested that the capacity to generate HS-anMan was either exhausted or not fully utilized. The HS-anMan clusters also stained for SYN (Fig. 7C, yellow in merged) and staining for SYNfil co-localized intensely with SYN staining indicating that much of the SYN was filamentous (Fig. 7D, yellow in merged).

**Fig. 8:**
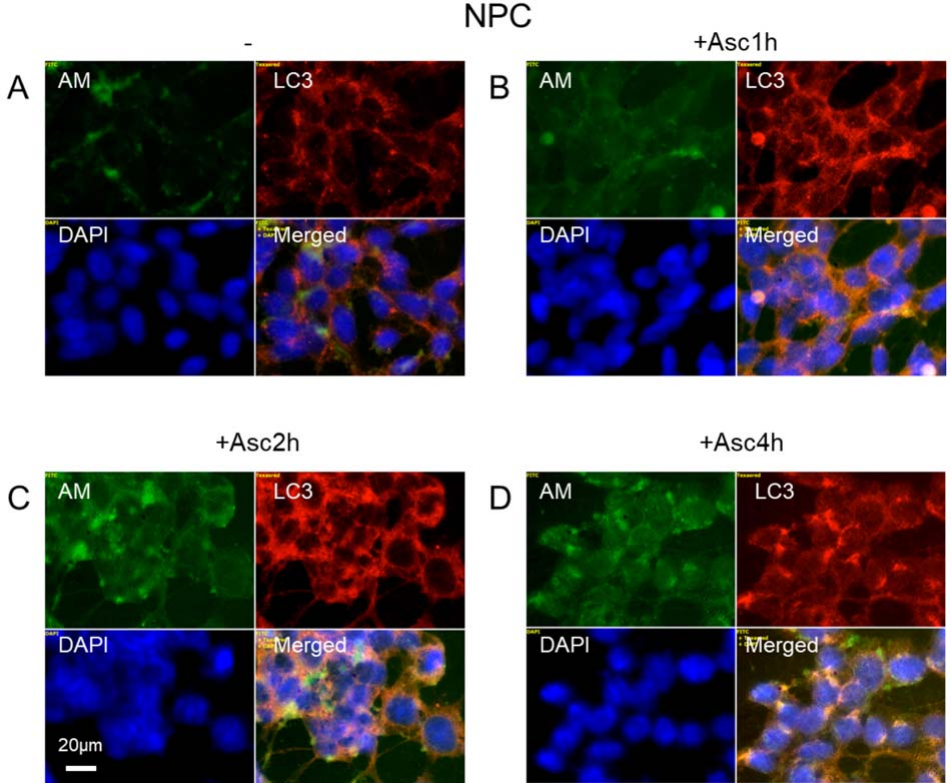
Ascorbate stimulates increased formation and clustering of AM-containing HS (HS-anMan) in human NPC. Representative immunofluorescence images of confluent cultures of NPC grown to confluence in regular medium and then treated with 1 mM ascorbate for the indicated periods of time. Staining was performed with mAb AM (for HS-anMan, green), pAb LC3 (for autophagosomes, red) and DAPI (for nuclei, blue). Exposure time was the same in all cases. Bar, 20 μm.

To examine if there was residual capacity to generate HS-anMan, NPC were treated with 1 mM ascorbate, which can initiate deaminative release of HS-anMan (Mani et al. 2006; Cheng et al. 2011, 2012, 2014, 2017). When the NPC cultures were examined at low magnification, a general increase of HS-anMan staining intensity was notable. The intensity of AM/intensity of DAPI increased almost 4-fold over a 4-h period (Supplementary Figure S5, AM, green and inset AM/DAPI). At high magnification, clustering of HS-anMan staining was observed over the same time period (Fig. 8A-D, AM, green). Some of the clusters were separate, but there was also co-localization with LC3 suggesting association with autophagosomes (Fig. 8C-D, both green and orange in merged). This indicates that NPC had a dormant capacity to generate HS-anMan, some of which was transported into the cytosol and captured by autophagosomes.

**Fig. 9:**
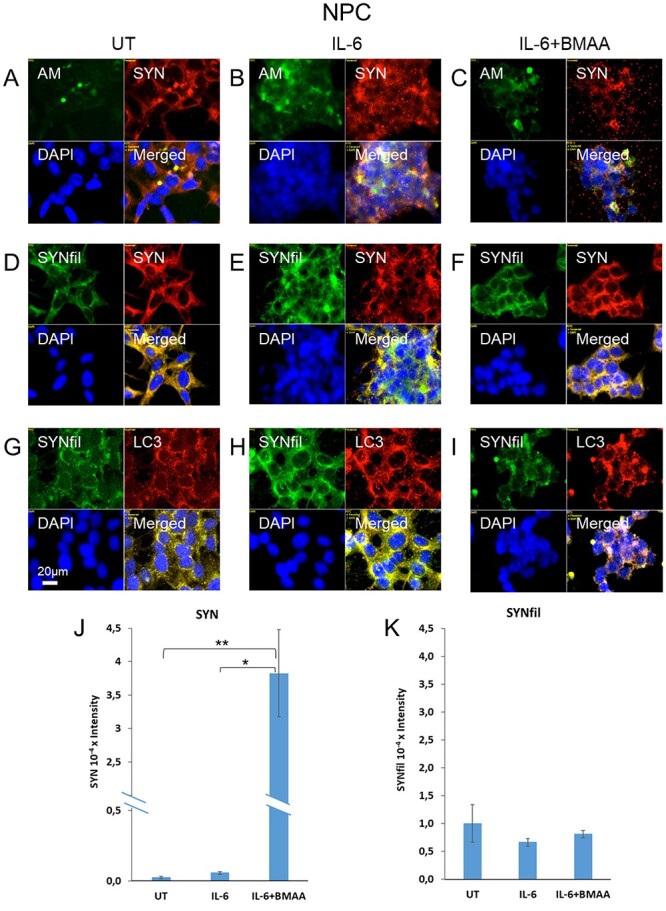
Treatment with β-methylamino-L-alanine (BMAA) decreases formation of AM-containing HS (HS-anMan), decreases accumulation of α-SYN aggregates in autophagosomes, and increases secretion of α-SYN in IL-6-treated human NPC. Representative immunofluorescence images of confluent cultures of NPC grown to confluence in regular medium (A, D, G, UT = untreated), or in medium containing 100 ng/ml IL-6 (B, E, H) or 100 ng/ml IL-6 and 15 mM BMAA (C, F, I). Staining was performed with mAb AM (for HS-anMan, green), pAb SYN (red), mAb SYNfil (green), pAb LC3 (for autophagosomes, red) and DAPI (blue, for nuclei). Exposure time was the same in all cases. Bar, 20 μM. Slot blot assay of SYN (J) and SYNfil (K) secretion. Medium volume, 2.8 ml; sample volume, 30 μL. The intensity of the controls (no primary antibody added) was subtracted. The error bars indicate S.E., *n* = 5; ^*^^*^, *P* < 0.1.

Next, we examined the effect of the cytokines TNF-α, IL-1β and IL-6 on SYN aggregation in NPC. In these cells, formation of SYNfil and co-localization with LC3 was most pronounced in IL-6-treated cells, followed by IL-1β (cf. Supplementary Figure S6A with Supplementary Figure S6C and Supplementary Figure S6D). TNF-α had almost no effect (cf. Supplementary Figure S6A with Supplementary Figure S6B). As cytokines increase the expression of β-secretase and thereby indirectly stimulate Gpc-1 processing, we examined IL-6-treated NPC for HS-anMan production. When the cells were grown in the presence of IL-6, there was increased clustered staining for HS-anMan that colocalized with SYN staining (Fig. 9A and B). The SYN staining also colocalized with staining for SYNfil and LC3 (Fig. 9D, E, G and H). The appearance of the AM/SYN clusters (Fig. 9B, merged) and the SYNfil/SYN clusters (Fig. 9E, merged) were partly similar to those seen in TNF-α-treated SH-SY5Y cells (green in the center, see Fig. 3C, merged).

To attenuate HS-anMan formation in IL-6-treated cells we used BMAA. This non-protein amino acid can replace some of the Ser residues in the attachment region for glycosaminoglycans, including the Gpc-1 core protein. Addition of HS is, therefore, precluded at these sites and a HS-depleted Gpc-1 is obtained (Cheng et al. 2019). When NPC were grown in the presence of both IL-6 and BMAA, there was reduction in the staining intensity, the extent of clustering, and colocalization with LC3 of both HS-anMan and SYNfil (Fig. 9C, F and I). We also assessed the extent of SYN and SYNfil secretion by slot blotting. While there was little secretion in untreated as well as IL-6-treated NPC, the presence of BMAA during the growth phase induced massive, almost 70-fold, increased secretion of SYN, in a non-aggregated state (Fig. 9J and K).

## Discussion

**Fig. 10:**
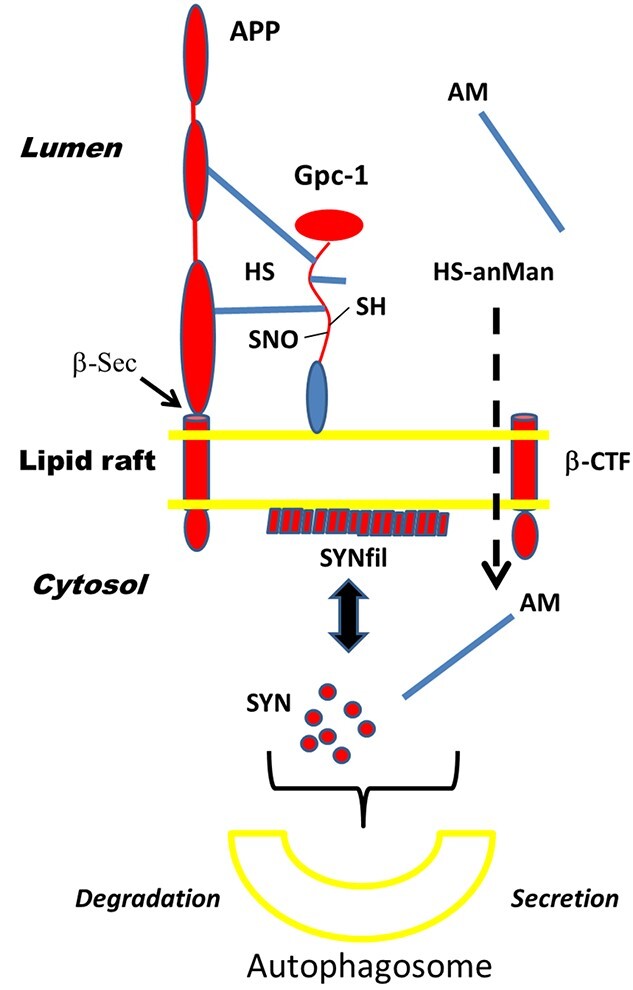
The topology of APP, Gpc-1, and α-SYN monomers (SYN) and filaments (SYNfil) associated with an endosomal lipid raft and possible fate of released heparan sulfate (HS) and SYN/SYNfil. β-Sec arrow, β-secretase cleavage site; HS, heparan sulfate chains; SH, thiol; SNO, S-nitrosothiol; HS-anMan, deaminatively released heparan sulfate, terminating with AM; β-CTF, C-terminal fragment of APP generated by β-secretase cleavage.

Gpc-1 can release its HS-chains by deaminative cleavage catalyzed by NO derived from SNO Cys residues located close to the HS attachment sites (Fig. 10). Neural cells may also express Gpc-2. As Gpc-2 lacks Cys residues in the C-terminal region it is not expected to contain SNO groups. Other Cys residues in Gpc-2 would be involved in disulfide bonds. However, minor Gpc forms, like Gpc-4, could potentially generate HS-anMan (Cheng et al. 2012).

In endosomes, Gpc-1 is attached to the luminal side of lipid rafts via its C-terminal GPI- anchor (Fig. 10). APP, a type I membrane-spanning protein, is also present in lipid rafts owing to its binding to Gpc-1, both via protein-to-protein and protein-to-HS interactions (Reinhard et al. 2005; Mani et al. 2006). Cleavage of APP by β-secretase releases a soluble N-terminal fragment (β-NTF), while the C-terminal fragment (β-CTF) remains attached to the membrane. This fragment can be further degraded by γ-secretase, yielding Αβ peptides. The HS-anMan released from Gpc-1 is transported through the membrane into the cytosol, possibly dependent on β-CTF and expression of the normal Niemann-Pick C1 protein. As Aβ peptides can aggregate and form membrane-penetrating pores, β-CTF may also form similar structures (Benilova et al. 2012; Larson and Lesné 2012; Cheng et al. 2017).

SYN is attracted to the cytosolic side of lipid rafts, which induces filamentous aggregation (SYNfil). As co-localization was observed between Gpc-1 and SYN/SYNfil in both SH-SY5Y and NPC cells, they may be located opposite to one another and only separated by the lipid membrane (Fig. 10). In SH-SY5Y cells, there was also partial co-localization between HS-anMan and SYN. Cytosolic HS-anMan is captured by autophagosomes and degraded in lysosomes (Cheng et al. 2014, 2017). Autophagosomal accumulation of SYN/SYNfil can lead to degradation or secretion (Fig. 10). HS-anMan is thus a potential modulator of SYN aggregation and/or capture by the autophagosomes).

**Fig. 11:**
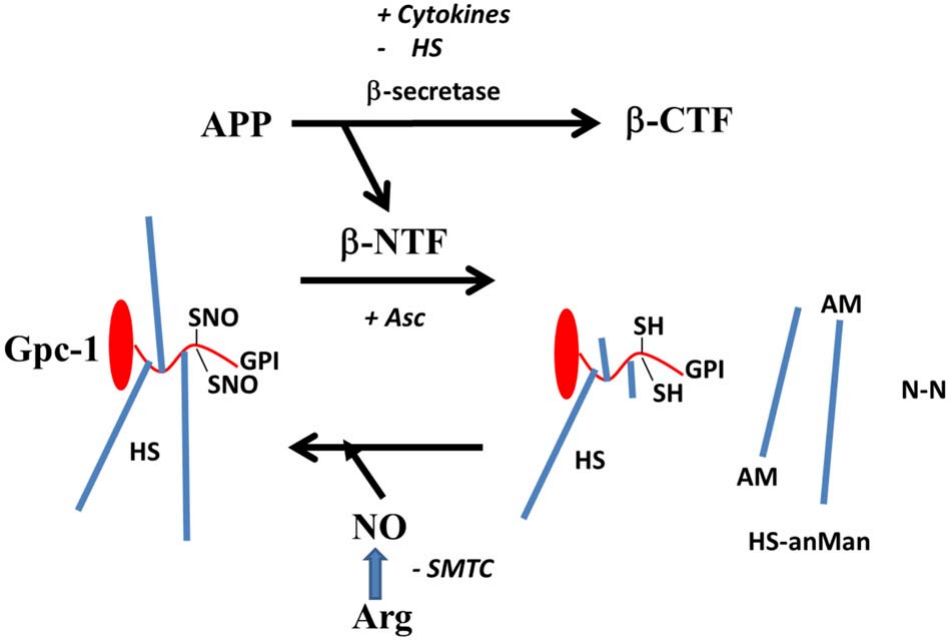
Processing and recycling of Gpc-1. The N-terminal fragment (β-NTF) generated by β-secretase cleavage of the APP initiates deaminative heparan sulfate (HS) release from Gpc-1. The products are a HS-truncated Gpc-1, HS with reducing terminal AM (HS-anMan), and molecular nitrogen (N-N). Re-nitrosylation by nitric oxide (NO) derived from Arg occurs after recycling of the HS-truncated Gpc-1 via the Golgi and re-endocytosis. β-CTF, C-terminal fragment of APP; SNO, S-nitrosothiol; SH, thiol; Asc, ascorbate; AM, anhydromannose; SMTC, S-methyl-L-thiocitrulline.

Cell-surface, lipid-raft-associated Gpc-1 is endocytosed, S-nitrosylated and transported to endosomes. After cleavage of the HS chains, the HS-truncated Gpc-1 recycles to the Golgi, where new HS chains are assembled, and then re-secreted to the cell-surface (Edgren et al. 1997; Cheng et al. 2002, 2012, 2014, 2017). Processing of Gpc-1-SNO is initiated by β-NTF, which is released from APP by β-secretase (Fig. 11). Expression of β-secretase is enhanced by proinflammatory cytokines, resulting in increased Gpc-1 processing (Cheng et al. 2020). β-Secretase activity is inhibited by HS (Scholefield et al. 2003), thereby constituting a negative feed-back loop. Exogenous ascorbate stimulates Gpc-1 processing via an electron transport chain involving endosomal cytochrome b561, which provides reducing power to fuel copper recycling in β-NTF (Cheng et al. 2017). Re-nitrosylation of the recycling Gpc-1 can be blocked by inhibiting NO-synthase by SMTC. In summary, cytokines, ascorbate and NO synergistically increase HS-anMan formation.

In SH-SY5Y cells, Gpc-1-SNO processing and regeneration appeared to be in a steady-state as TNF-α had only a minor effect on HS-anMan production. The major rate-limiting factor appeared to be the low level of S-nitrosylation in the Gpc-1 protein. This could be corrected by supplementation with Arg (precursor of NO). Overexpression of Gpc-1 alone had a limited effect, as both the exogenous and the endogenous Gpc-1 are dependent on supply of NO for the release of HS-anMan.

When SH-SY5Y cells were grown in the presence of TNF-α, there was increased clustering of SYNfil and HS-anMan and an enlargement of the autophagosomes, which could be a cytokine effect on APP processing resulting in accumulation of β-CTF (Cheng et al. 2020). When Arg was included in the growth medium in order to increase HS-anMan formation, accumulation of both SYNfil and HS-anMan disappeared. As there was no sign of greatly increased secretion, we assume that they were degraded in the lysosomes. We also examined the effect of TNF-α when formation of NO was attenuated and HS-anMan formation therefore suppressed. Similarly, the clustered accumulation of SYNfil/SYN disappeared. However, now there was increased secretion of SYN. Thus, HS-anMan formation and degradation may be critical for efficient autophagy of SYN. As shown elsewhere, HS-anMan degradation products inhibit Aβ oligomerization (Cheng et al. 2011). It has been reported that NO can inhibit autophagosome synthesis (Sarkar et al. 2011). In our study, increased NO production did not appear to prevent autophagy of SYN, while inhibition of NO production resulted in increased secretion.

In NPC, Gpc-1 processing was limited, little HS-anMan was generated and much of the SYN was in a filamentous state. However, when exogenous ascorbate was added, formation of HS-anMan was initiated and it was rapidly transported to autophagosomes. This latent state could be due to inhibition of β-secretase by HS, which was then circumvented by activation of copper recycling in APP β-NTF by ascorbate. Moreover, formation of HS-anMan is accompanied by endosomal acidification, which stimulates β-secretase (Cheng et al. 2021).

When NPC were grown to confluence in the presence of IL-6, HS-anMan formation was activated and SYN aggregates and HS-anMan accumulated. Gpc-1 normally carries 3 HS chains, but cells grown in the presence of BMAA produce a single HS-chain Gpc-1 and the capacity to generate HS-anMan is therefore reduced (Cheng et al. 2019). The single HS chain may protect the core protein from unfolding (Svensson et al. 2009). As the Gpc-1 protein is recycled and only HS-chains are degraded and re-assembled on the remaining stubs, the single-chain HS substitution should persist during the growth-phase (Edgren et al. 1997). Therefore, when NPC were grown in the presence of both IL-6 and BMAA, less HS-anMan was formed and non-aggregated SYN was secreted. When non-dividing neural cells or neurons are exposed to BMAA, there is no effect on HS-anMan formation or on APP processing (Cheng et al. 2019). Nevertheless, it cannot be excluded that other cell-associated, but non-recycling, HS-deficient proteoglycans contribute to the paucity of HS. We suggest that free HS chains may play a role in SYN aggregation and for the degradation of SYN/SYNfil in autophagosomes/lysosomes (Fig. 11).

The cytokine-induced clustering of SYN was conspicuously similar to the previously observed effects on APP processing in dividing mouse N2a and human stem cells (Cheng et al. 2020). Here, the cytokines induced accumulation of complexes between HS-anMan and APP β-CTF in enlarged autophagosomes/lysosomes. Moreover, the clustered accumulation of β-CTF disappeared when HS-anMan release was suppressed by NO-deprivation.

Dietary intake of the cyanobacterial neurotoxin BMAA is believed to cause a mixed AD-PD neuropathology (Cox et al. 2018). As exposure to BMAA leads to formation of HS-deficient Gpc-1 (Cheng et al. 2019), the reduced capacity to generate HS-anMan should result in increased processing of APP due to decreased inhibition of β-secretase by HS. Consequently, APP β-CTF may accumulate in autophagosomes.

In many dementia cases, there are also mixed AD-PD neuropathological features and Aβ and SYN peptides occur together in both amyloid plaques and Lewy bodies. In vitro studies have shown that Aβ peptides can trigger aggregation of SYN (Köppen et al. 2020). As Aβ constitutes the N-terminal domain in β-CTF, the latter may also be a trigger of SYN aggregation in vivo.

The apolipoprotein E4 (ApoE4) genotype is the most influential risk factor for sporadic AD. We have recently investigated APP and Gpc-1 processing in ApoE4-expressing human fibroblasts and in induced neurons obtained by reprogramming of ApoE4 fibroblasts (Cheng et al. 2021). In the ApoE4 fibroblasts, the capacity to generate HS-anMan was not fully utilized. In the fibroblast as well as in the induced neurons, there was accumulation of APP degradation products (β-CTF and Aβ). This was reversed when increased formation of HS-anMan was induced by ascorbate or dehydroascorbate. Interestingly, the ApoE4 genotype also promotes synucleinopathy (Fyfe 2020 and refs. therein).

There is a mutual functional relationship between APP and Gpc-1. We suggest a scenario, which begins with cleavage of APP by β-secretase. This releases β-NTF, which initiates release of HS-anMan from Gpc-1. HS-anMan is an inhibitor of β-secretase, whereby the generation of APP degradation products is moderated. This balance is disturbed by proinflammatory cytokines, which increase the capacity for β-cleavage of APP. When the inhibition by HS-anMan becomes insufficient, complexes between HS-anMan and β-CTF accumulate. The latter and/or Aβ, then triggers SYN aggregation. If HS-anMan production is attenuated under these conditions, by HS- or NO-deficiency, autophagy is hampered and both Aβ and SYN are secreted. Hence, both can then accumulate together in AD plaques as well as Lewy bodies. Also HS-anMan can be detected in AD plaques (Cheng et al. 2011).

## Materials and methods

### Cells and reagents

Human neuronal progenitor cells (NPC) were purchased from ATCC, USA, plated in CellMatrix gel-coated plates and maintained as recommended by the supplier. Human neuroblastoma SH-SY5Y cells were a gift from Professor Jia-Yi Li, Lund University. The cells were cultured in a 1:1 mixture of Eagle’s Minimum Essential Medium and F12 Medium supplemented with 10% (v/v) fetal bovine serum according instructions provided by ATCC. Cells were transiently transfected with a vector encoding green fluorescent protein (GFP)-tagged mouse Gpc-1 protein as described previously (Cheng et al. 2011). The coding sequence included an N-terminal signal peptide, the GFP with a disrupted start codon, followed by the Gpc-1 core protein including the C-terminal signal peptide for membrane attachment. TNF-α was from Alomone labs, Israel, IL-1β from Gibco, Sweden and IL-6 from Tataabiocenterr, Sweden). Mouse anti-α-SYN (mAb SYN) was obtained from BD biosciencesr, Sweden (610786) and rabbit anti-α-SYN (pAb SYN) from Chemicon, Sweden (ab5038). A rabbit monoclonal against α-SYNfils (mAb SYNfil) was purchased from Abcam, UK (MJFR-14-6-4-2, ab209538). mAb AM is specific for HS/heparin tetrasaccharide or larger fragments that are generated by partial deaminative cleavage and therefore terminate with anhydromannose (anMan) at the reducing end (Pejler et al. 1988). In Gpc-1-HS, the in vivo cleavage occurs at N-unsubstituted glucosamines (Cheng et al. 2012). Staining with mAb JM-403, which recognizes the cleavage sites, disappears when cells are treated with ascorbate. Concomitantly, staining with mAb AM appears (Ding et al. 2002). Similarly, staining with another HS-specific mAb (10E4) disappears upon ascorbate treatment with simultaneous appearance of mAb AM staining (Mani et al. 2004). A rabbit antiserum raised against human Gpc-1 has been described earlier (Mani et al. 2006) as well as a mAb S1 recognizing Gpc-1-SNO (de Boeck et al. 1987; Cheng et al. 2002; Mani et al. 2003). Polyclonal anti-Rab7 (sc107867) was from Santa Cruz, USA and anti-LC3 and FITC-tagged goat anti-mouse Ig from Sigma-Aldrich, Sweden. Alexa-Fluor 594-tagged donkey anti-rabbit IgG was from Invitrogen, Sweden, Alexa Fluor 594-tagged goat anti-mouse IgG and OregonGreen 488-tagged goat anti-rabbit IgG from Molecular Probes, USA and Dynabeads M-280 sheep anti-rabbit IgG from Thermo Fisher Scientific, Sweden. The commercial antibodies were used as recommended by the manufacturers. The DNA staining compound 4,6-diaminido-2-phenylindole (DAPI), β-N-methylamino-L-alanine (BMAA), sodium nitroprusside (SNP) and ascorbate were obtained from Sigma-Aldrich, Sweden. S-Methyl-L-thiocitrulline (SMTC) was purchased from Santa Cruz, USA.

### Deconvolution immunofluorescence microscopy

Cells were examined by immunofluorescence microscopy as described previously (Cheng et al. 2014). In brief, cells were fixed in acetone in order to retain cellular and subcellular structures and to ensure the preservation of carbohydrates. The fixed cells were first pre-coated with 10% anti-mouse total Ig and then exposed to primary antibodies overnight. The secondary antibodies used were FITC-tagged goat antimouse Ig or OregonGreen 488-tagged goat anti-rabbit IgG when the primary antibody was a monoclonal and Alexa Fluor 594-tagged goat anti-rabbit IgG or sometimes Alexa Fluor 594-tagged donkey anti-goat IgG when the primary antibody was a polyclonal. In the controls, the primary antibody was omitted. DNA staining with (DAPI), as well as staining with antibodies was performed as recommended by the manufacturers. The fluorescent images were analyzed by using a Carl Zeiss AxioObserver inverted fluorescence microscope with deconvolution technique and equipped with objective EC “Plan-Neofluar” 63×/ 1.25 Oil M27 and AxioCam MRm Rev Camera. Identical exposure settings and times were used for all images. During microscopy, the entire slides were scanned and immunofluorescence images at 20× and 100× magnifications were captured. The low magnification images were used to identify representative locations for high magnification images. For colocalization analysis, the fluorophores were excited in a sequential manner using multi-track acquisition to minimize cross-talk. Data analysis was performed using Zeiss AxioVision Release 4.8 software. Entire images were used for estimation of the extent of co-localization, which was expressed as the Pearson R value.

### Isolation of autophagosomes

Cells were homogenized in PBS containing 0.25 M sucrose, 0.1 M EDTA and 0.5 mM phenylmethyl sulfonyl fluoride at 4°C. Autophagosomes were isolated from the homogenates by using pAb LC3 as primary antibody and Dynabeads M-280 sheep anti-rabbit IgG as magnetic secondary antibody. The magnetic particles were recovered using a magnetic particle concentrator and extensively washed with PBS. The autophagosomes were lysed in 0.15 M NaCl, 10 mM EDTA, 2% (v/v) Triton X-100, 10 mM KH_2_PO_4_, pH 7.5.

### Slot blot

Membranes were probed with mAbs AM, SYN or SYNfil followed by visualization using horseradish peroxidase-conjugated goat anti-mouse or anti-rabbit IgG and developed by chemiluminescence (GE Healthcare, Sweden) using a Fujifilm detector. In the controls, the primary antibody was omitted. Intensity was measured by densitometry.

### Statistical methods

For statistical analysis, two group comparisons were performed using unpaired two tailed student *t*-test and unequal variances data analysis. Error probabilities of *P* < 0.05 were considered statistically significant. Indication of *P*-value summaries: **P < 0.01. Pearson’s correlation coefficients were used to analyze the extent of co-localizations.

## Abbreviations

anMan, anhydromannose; APP, amyloid precursor protein; AD. Alzheimer’s disease; BMAA, β-methylamino-L-alanine; CTF, C-terminal fragment; GFP, green fluorescent protein; Gpc-1, glypican-1; GPI, glycosyl-phosphatidyl-inositol; HS, heparan sulfate; IL-1β, interleukin-1-beta; IL-6, interleukin-6; mAb, monoclonal antibody; NO, nitric oxide; NTF, N-terminal fragment; pAb, polyclonal antibody; PD, Parkinson’s disease; SNO, S-nitrosothiol; SYN, α-SYN; SYNfil, filamentous SYN; TNF-α, tumor necrosis factor-alpha.

## Supplementary Material

Supplementary_material_cwab126
