# Supplementary material for: Complex modulation of cytokine-induced α-synuclein aggregation by glypican-1-derived heparan sulfate in neural cells
Source: Glycobiology. 2021 Dec 7;32(4):333–42. doi: 10.1093/glycob/cwab126 (PMC8970428; doi:10.1093/glycob/cwab126)
Supplement: Supplementary_material_cwab126 [file supplementary_material_cwab126.pdf]

Supplementary material

Complex modulation of cytokine-induced α-synuclein aggregation by glypican-1-derived heparan sulfate in neural cells

Key words: Glypican-1/ Heparan sulfate/ Nitric oxide/ Parkinson´s disease/ α-Synuclein

Running head: Modulation of α-synuclein aggregation by heparan sulfate

Fang Cheng, Lars-Åke Fransson and Katrin Mani*

Department of Experimental Medical Science, Division of Neuroscience, Glycobiology Group, Lund University, Biomedical Center A13, SE-221 84 Lund, Sweden

∗To whom correspondence should be addressed: Tel: +46-46-222-4044; e-mail: katrin.mani@med.lu.se

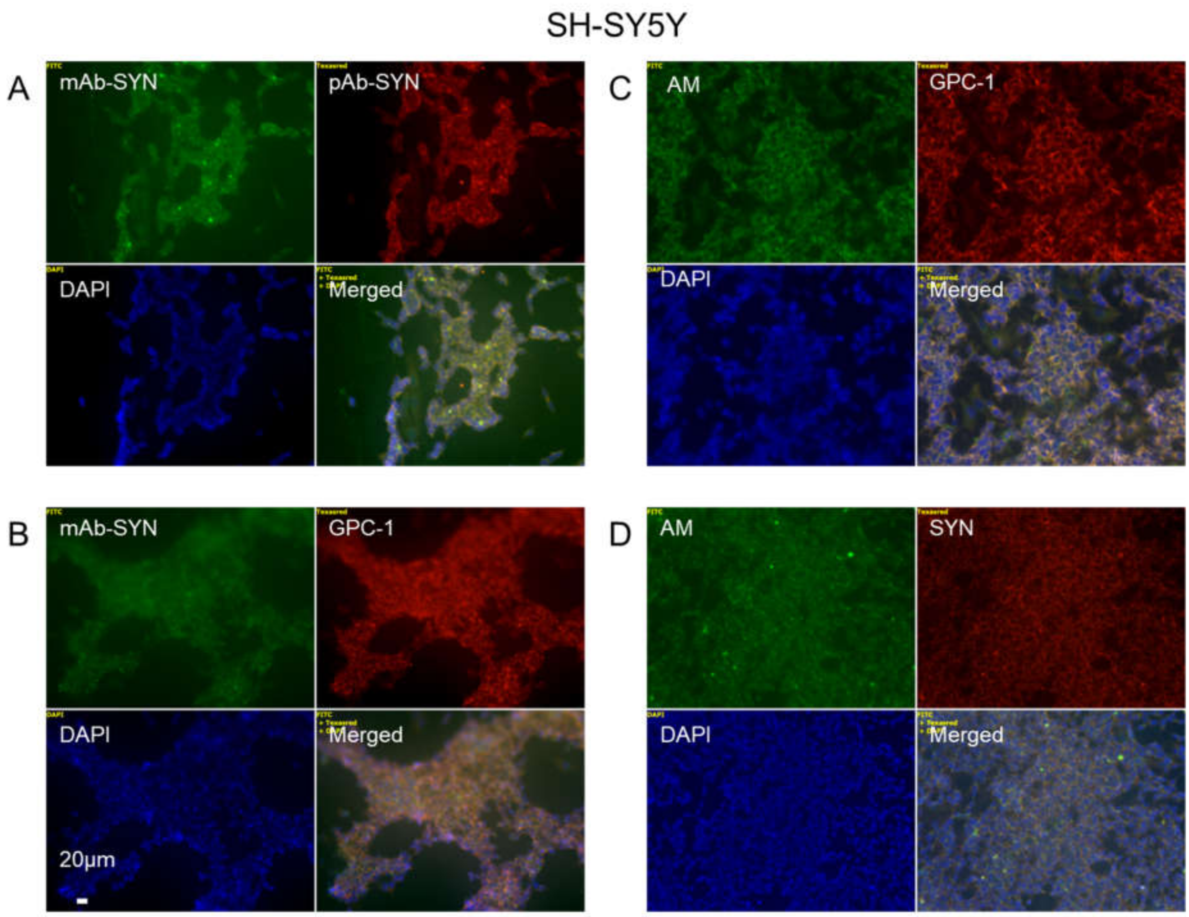

Fig.S1

Fig. S1. α-Synuclein (SYN) co-localizes with glypican-1 (Gpc-1) and anhydromannose-containing heparan sulfate (HS-anMan) in human neuroblastoma SH-SY5Y cells. Immunofluorescence images at low magnification of cell cultures grown to near confluence in regular medium. Staining was performed with mAb SYN (green), pAb SYN (red), pAb GPC-1 (red), mAb AM (for HS-anMan) and DAPI (for nuclei, blue). Exposure time was the same in all cases. Bar, 20 µm.

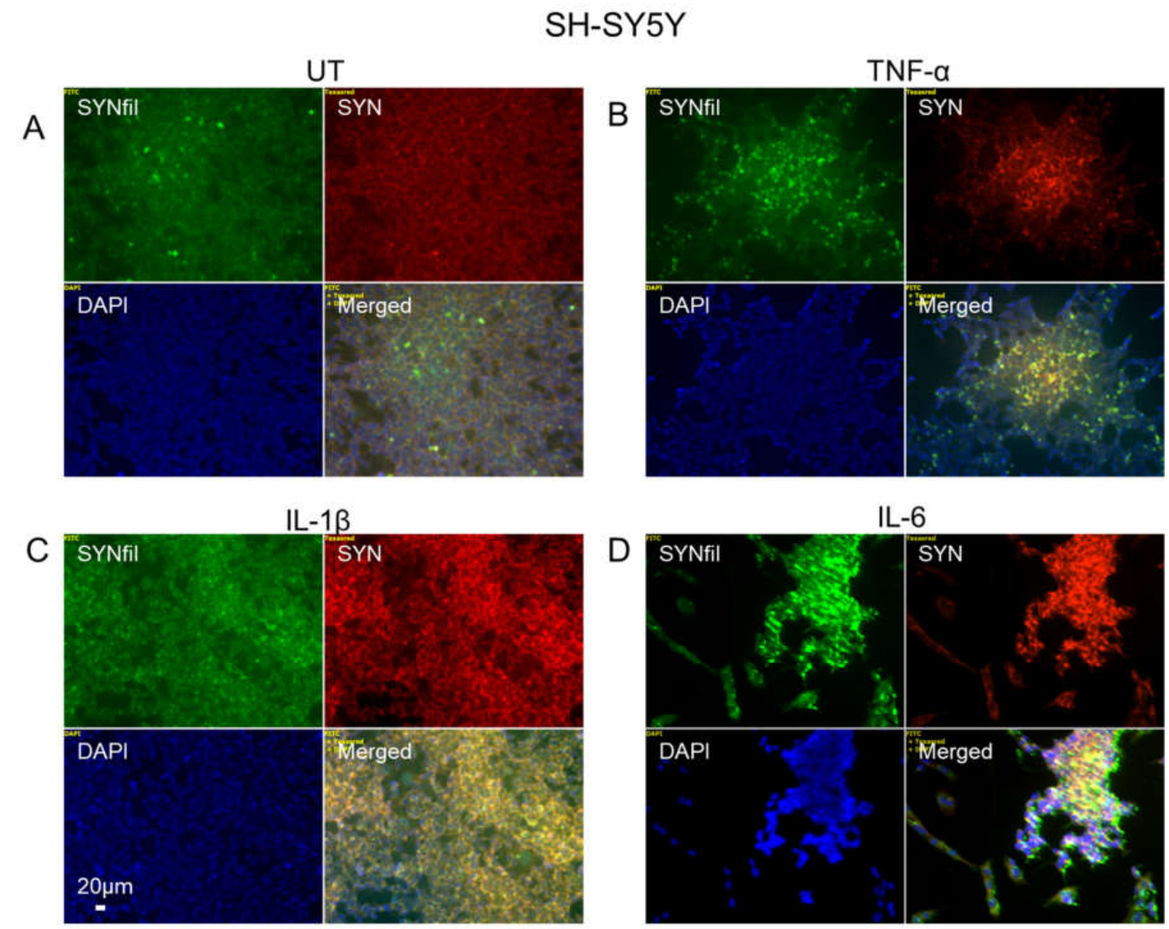

Fig.S2

Fig, S2. The cytokines TNF-α, IL-1β and IL-6 induce increased co-localization of α-synuclein filaments (SYNfil) and α-synuclein (SYN) in SH-SY5Y cells. Immunofluorescence images of confluent cultures of SH-SY5Y cells at low magnification. Cells were grown to confluence in regular medium (A, UT = untreated) or in medium containing 100 pg/ml TNF-α (B), 50 ng/ml IL-1β (C) or 100 ng/ml IL-6 (D). Staining was performed with mAb SYNfil (green), pAb SYN (red) and DAPI (for nuclei, blue). Exposure time was the same in all cases. Bar, 20 µm.

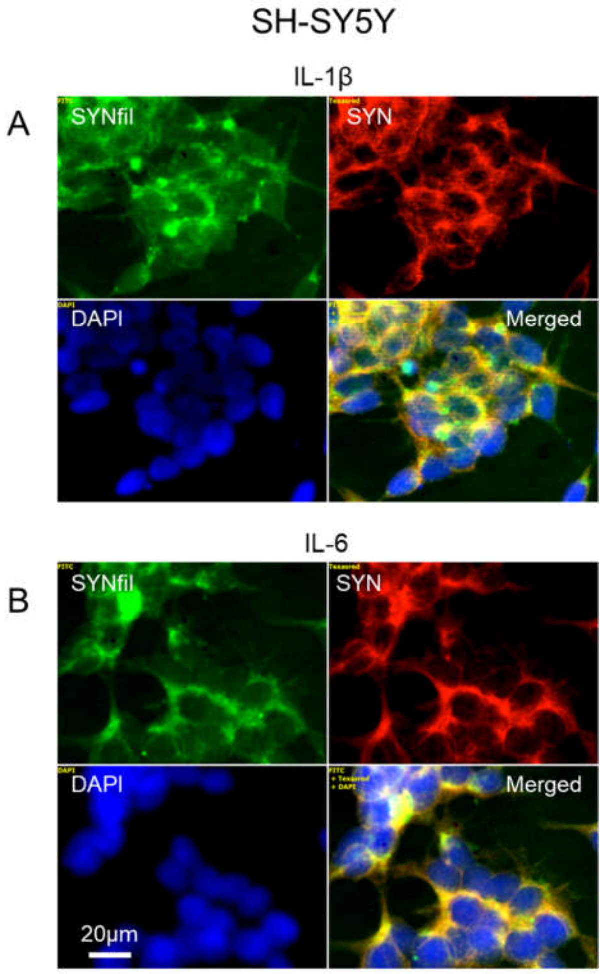

Fig.S3

Fig. S3. Clustered and diffuse co-localization of α-synuclein filaments (SYNfil) with α-synuclein (SYN) in SH-SY5Y cells upon growth in the presence of IL-1β (A) or IL-6 (B). Representative immunofluorescence images of confluent cultures of SH-SY5Y cells which were grown to confluence in medium containing 50 ng/ml IL-1β or 100 ng/ml IL-6. Staining was performed with mAb SYNfil (green), pAb SYN (red) and DAPI (for nuclei, blue). Exposure time was the same in both cases. Bar, 20 μm.

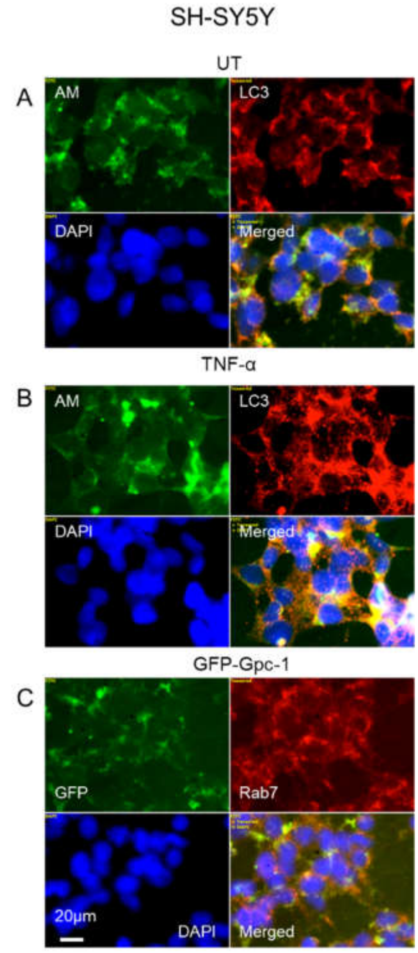

Fig.S4

Fig. S4. Tumor necrosis factor-α (TNF-α) induces slightly increased staining for anhydromannose-containing HS (HS-anMan) but no increased co-localization with the autophagosome marker LC3 in SH-SY5Y cells (A, B) and green fluorescent protein-tagged glypican-1 (GFP-Gpc-1) appears in Rab7-positive late endosomes (C). Representative immunofluorescence images of confluent cultures of SH-SY5Y cells which were grown to confluence in regular medium (A, UT=untreated) or in medium containing 100 pg/ml TNF-α (B) or transfected with a vector encoding GFP-tagged Gpc-1 (C). Staining was performed with mAb AM (for HS-anMan, green), a pAb against LC3 (autophagosome marker, red), and DAPI (for nuclei, blue) in A and B, and with a pAb against Rab7 (red) and DAPI (for nuclei, blue) in C. In C, GFP-Gpc-1 is visualized in the green channel. Exposure time was the same in all cases. Bar, 20 µm.

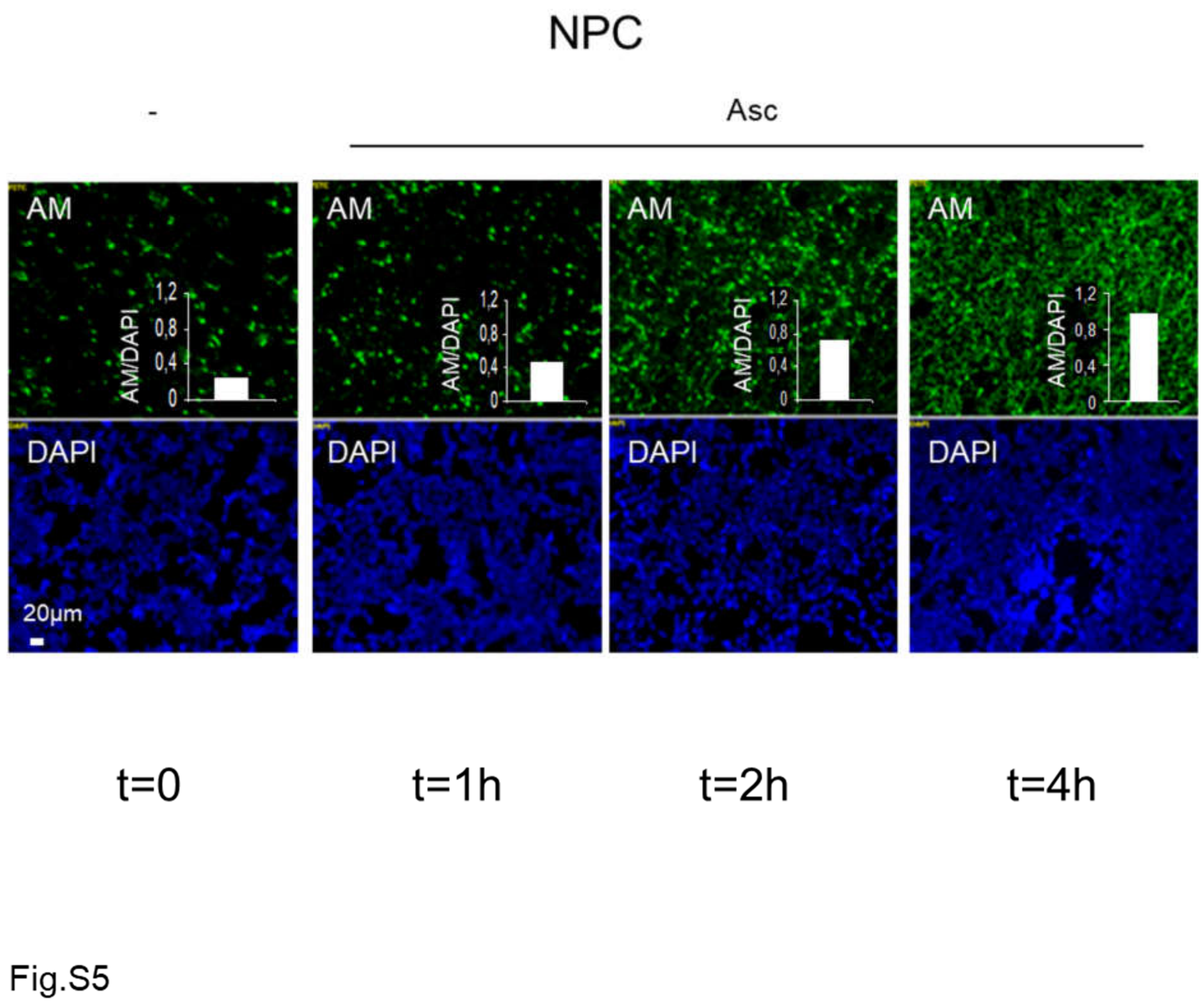

Fig. S5. Ascorbate induces increased release of anhydromannose-containing HS (HS-anMan) in neural progenitor cells (NPC). Representative immunofluorescence images of confluent cultures of NPC grown to confluence in regular medium and then treated with 1 mM ascorbate for the indicated periods of time. Staining was performed with mAb AM (for HS-anMan, green) and DAPI (for nuclei, blue). Exposure time was the same in all cases. Bar, 20 µM. Inset in AM: Intensity of HS-anMan staining (AM) divided with intensity of DAPI staining (white bars).

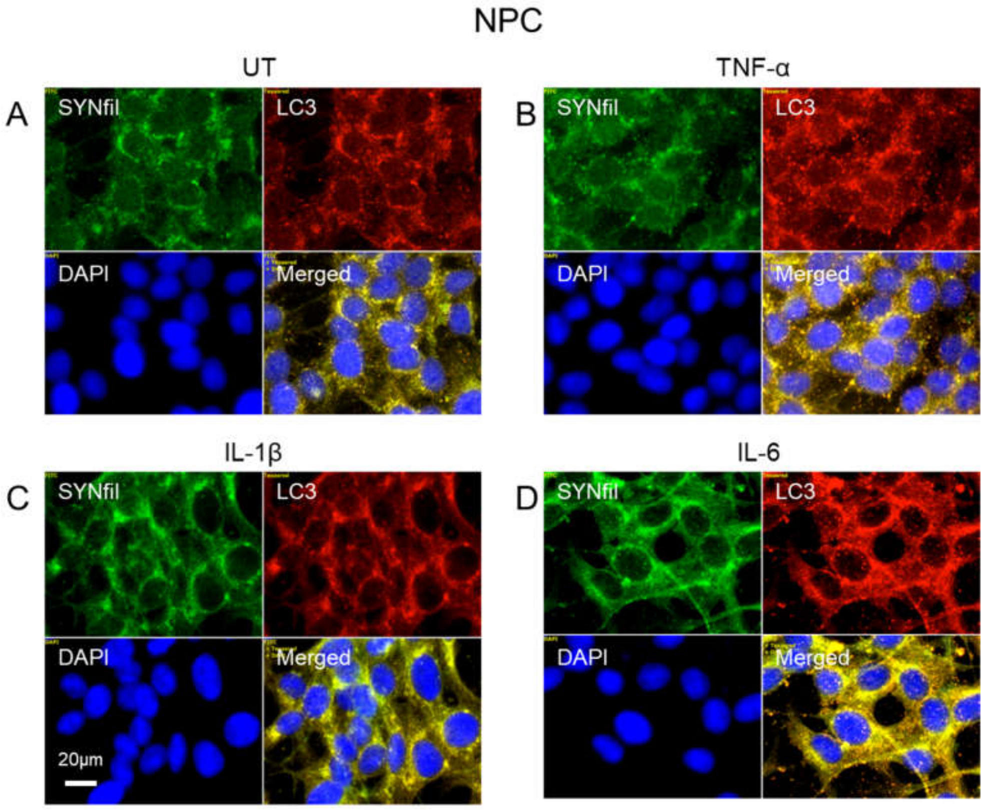

Fig. S6

Fig. S6. Effect of the cytokines on SYN aggregation and association with autophagosomes. Representative immunofluorescence images of confluent cultures of NPC grown to confluence in regular medium (A, UT=untreated), in medium containing 100 pg/ml TNF-α (B) or 50 ng/ml IL-1β (C) or 100 ng/ml IL-6 (D). Staining was performed with mAb SYNfil (green), pAb LC3 (red) and DAPI (for nuclei, blue). Exposure time was the same in all cases. Bar, 20 μM.
